# At the edge of viability: neonatologists’ moral experience under uncertainty in Türkiye

**DOI:** 10.1186/s12910-026-01466-8

**Published:** 2026-06-12

**Authors:** Gülten Çevik Nasırlıer, Rahime Aydın Er

**Affiliations:** 1 Department of Administrative Affairs, Kocaeli Provincial Health Directorate, Kocaeli, Türkiye; 2 Department of History of Medicine and Ethics, Kocaeli University School of Medicine, Kocaeli, Türkiye; 3 Department of Psychiatric Nursing, Kocaeli University Faculty of Health Sciences, Kocaeli, Türkiye

**Keywords:** Neonatology, Periviability, Moral distress, Moral loneliness, Ethical decision-making, Qualitative research

## Abstract

**Background:**

Clinical decision-making in periviability, typically between 22 and 25 weeks of gestation, represents one of the most complex intersections of medicine and ethics. Characterised by profound prognostic uncertainty and the absence of clear ethical consensus, this period places neonatologists under significant moral, emotional, and institutional pressure. This study explores how neonatologists in Türkiye experience ethical challenges when making clinical decisions for infants born at the threshold of viability.

**Methods:**

A qualitative phenomenological design was employed. Between March 2022 and June 2023, in-depth semi-structured interviews were conducted with 15 neonatologists working in tertiary and quaternary neonatal intensive care units across different regions of Türkiye. Data collection continued until data saturation was reached. All interviews were transcribed and analysed using thematic analysis.

**Results:**

Five interrelated themes were identified that characterise neonatologists’ moral experiences in periviable care: moral decision-making under prognostic uncertainty; moral distress in perceived non-beneficial treatment; moral tensions in clinician–parent interactions; legal and institutional constraints on ethical decision-making; and moral loneliness in periviable care. Together, these themes highlight the ethical complexity of caring for infants at the threshold of viability, where uncertainty, responsibility, relational tensions, and structural pressures intersect.

**Conclusion:**

In neonatal intensive care settings in Türkiye, periviable care is experienced as an ethically complex practice shaped by clinical uncertainty and institutional conditions. Strengthening institutional mechanisms for ethical deliberation may help reduce clinicians’ experiences of moral loneliness.

**Supplementary Information:**

The online version contains supplementary material available at 10.1186/s12910-026-01466-8.

## Introduction

Clinical and ethical decision-making at the threshold of periviability, commonly defined as 22 to 25 weeks of gestation, represents one of the most ethically demanding areas of neonatal medicine [1]. At this boundary, survival remains uncertain and the risk of severe neurodevelopmental impairment is substantial, requiring time-sensitive decisions under conditions of profound prognostic ambiguity [2]. Under such conditions, clinical judgment cannot be reduced to biomedical indicators alone; rather, it is closely linked to ethical reasoning, professional responsibility, and accountability for serious clinical consequences [3].

To navigate this uncertainty, many high-income countries have introduced the concept of a “grey zone,” referring to gestational periods in which active resuscitation, comfort care, or structured parental involvement in decision-making may be variably prioritised [1, 4, 5]. Although such frameworks provide normative orientation, they do not fully capture how clinicians experience the moral weight of acting under uncertainty in real-time practice [6, 7].

Within neonatal ethics literature, clinicians’ ethical strain in these situations is most frequently conceptualised as moral distress [8–10]. Moral distress is commonly defined as the psychological and ethical burden that arises when clinicians are constrained from acting in accordance with what they judge to be ethically appropriate [11, 12]. However, empirical research suggests that ethical strain in periviable care is not limited to situations of overt constraint [9, 10, 12]. Clinicians also report forms of moral unease, characterised by doubt, regret, and persistent self-questioning in situations where decisions are ethically defensible yet prognostically uncertain [8, 10, 13]. Distinguishing between constraint-based moral distress and uncertainty-related moral unease is important, as it underscores that ethical difficulty in periviable care may arise both from external pressures and from the intrinsic ambiguity of prognosis itself.

Empirical studies from Europe and North America demonstrate that such ethical strain persists even in contexts with established shared decision-making models and detailed professional guidance [6, 9, 14, 15]. For infants born at 22–24 weeks’ gestation, resuscitation is often initiated provisionally, while decisions about the continuation or withdrawal of life-sustaining treatment are deferred until postnatal clinical assessment [14, 15]. When outcomes are poor, clinicians frequently describe distress framed as having done “too much” or “not enough” for the infant [8, 10]. These findings suggest that moral strain arises not only from uncertainty but from assuming responsibility for consequential decisions under uncertainty.

Increasingly, moral distress in periviable care is understood as shaped by structural conditions, including legal frameworks, organisational cultures, hierarchical dynamics, and access to ethics consultation [6, 10, 16, 17]. Studies from Greece describe ethical strain emerging both from external constraints and from persistent uncertainty in prognostic decision-making [8, 18]. Research from Iran similarly highlights moral distress in situations where clear, consistent, and binding national guidance for periviability decision-making is absent [17, 19, 20]. In such settings, tensions between religious norms, clinical judgment, and fear of legal repercussions further intensify clinicians’ ethical vulnerability.

Within this international landscape, Türkiye represents a distinctive regulatory and institutional setting. Periviable care takes place within a legal framework in which the withdrawal or withholding of life-sustaining treatment is not explicitly regulated. Under this framework, end-of-life decisions are not governed by provisions specific to neonatal care but may be considered under general provisions of the Turkish Penal Code. Article 81 regulates intentional killing and applies to acts involving direct causation of death. In contrast, Article 83 addresses causing death through omission, stating that failure to perform an act that one is legally obliged to carry out may result in criminal liability if this omission leads to death [21].

The legislation does not explicitly address how these provisions apply to the limitation of life-sustaining treatment in cases of extreme prematurity, nor does it provide specific guidance for end-of-life decision-making in neonatal care. In addition, administrative regulations require that any infant showing signs of life be registered as a live birth regardless of gestational age [22]. Professional guidelines addressing neonatal care are available [23]; however, there is no binding national framework that explicitly regulates ethically complex decision-making at the threshold of viability. A national protocol defining neonatal intensive care unit admission and discharge criteria is in place [24], but this document primarily focuses on organisational levels of care and clinical admission indications.

Moreover, the available clinical evidence regarding periviable outcomes in Türkiye further complicates ethical deliberation. Most available studies from Türkiye examine broader populations of extremely or very low birth weight infants rather than focusing specifically on periviable gestational ages. Despite this limitation, available studies suggest that survival at the threshold of viability remains low and varies markedly by gestational age. For example, no infants born at 22 weeks survived to discharge in one cohort, while survival among those born at 23 weeks was reported to be 7.5%, increasing to 29.1% at 24 weeks [25]. In another study including infants born at 22–24 weeks, overall survival was reported as 21.2% [26]. Regional and multicentre studies also document substantial morbidity among survivors, including severe intraventricular haemorrhage, late-onset sepsis, bronchopulmonary dysplasia, and treatment-requiring retinopathy of prematurity [27, 28]. However, Türkiye lacks recent multicentre cohort data integrating gestational age-specific survival with standardised long-term neurodevelopmental outcomes for infants born at the threshold of viability. The coexistence of high mortality, significant morbidity among survivors, and the absence of comprehensive longitudinal outcome data sustains prognostic uncertainty. Such epistemic uncertainty may intensify neonatologists’ ethical vulnerability and complicate parental counselling at the limits of viability.

Despite these clinical and contextual challenges, research in Türkiye has predominantly focused on survival and morbidity outcomes [25–28]. The moral experiences of neonatologists, particularly how they interpret and navigate ethical challenges in periviable care within this regulatory framework, remain insufficiently explored. This study aimed to explore how neonatologists in Türkiye experience ethical dilemmas, moral distress, and professional responsibility in decision-making at the threshold of viability, and how these experiences are shaped by prognostic uncertainty and institutional constraints.

## Methods

### Study design

This article reports data derived from a doctoral dissertation examining ethical issues encountered by neonatologists in clinical decision-making for extremely preterm infants. A qualitative phenomenological design was employed to foreground neonatologists lived moral experiences and to explore how ethical challenges are experienced under conditions of prognostic uncertainty, time pressure, and institutional constraints [29]. The study is reported in accordance with the Consolidated Criteria for Reporting Qualitative Research (COREQ) checklist to ensure clear and comprehensive reporting [30] (Appendix 1).

### Participants and sampling

Neonatal care in Türkiye is organised across four levels of neonatal intensive care units (NICUs) [24] and is delivered within a heterogeneous institutional landscape including university hospitals, training and research hospitals, and private healthcare facilities. According to a recent report by the Turkish Medical Association, 391 neonatologists were actively practising in Türkiye in 2024 [31]. In some centres located in rural regions, clinical responsibility for periviable care may be shared with paediatricians due to workforce distribution and institutional capacity constraints.

Purposive sampling was used to capture diverse perspectives on periviable decision-making [29]. Participants were recruited from tertiary and quaternary neonatal intensive care units across different institutional settings and geographic regions. Inclusion criteria were practising as a neonatologist and involvement in clinical decision-making regarding the management of infants born at 22–25 weeks’ gestation. Neonatologists who did not volunteer to participate in the study were excluded.

Participant recruitment followed a two-stage strategy. First, an invitation letter describing the study was circulated via the official mailing list of the Turkish Neonatology Society and distributed to neonatologists who were members of the society. Second, snowball sampling was employed to identify additional participants; interviewees were invited to recommend colleagues who met the eligibility criteria and had experience with decision-making for infants born at the threshold of viability [29].

Consistent with qualitative research standards, no predetermined sample size was established [32]. Recruitment and analysis proceeded concurrently and continued until thematic saturation was achieved, defined as the point at which successive interviews yielded no substantively new codes or conceptual insights [33]. The determination of saturation was made collaboratively by both researchers through iterative analytical discussions. After the fifteenth interview, the researchers agreed that no new experiential dimensions were emerging and that thematic categories had achieved sufficient conceptual depth; recruitment was therefore discontinued. In addition, the assessment of data saturation was periodically discussed during doctoral thesis monitoring committee meetings.

### Data collection

Data were collected between March 2022 and June 2023 through semi-structured, in-depth interviews. The interview guide was developed based on the perinatal ethics literature and relevant empirical studies [6, 9, 34] and was pilot tested with two neonatologists to ensure conceptual clarity and contextual relevance. These participants were not members of the research team, and the pilot interviews were retained in the dataset because no substantial modifications to the guide were required. Minor refinements were introduced during the research process to enhance clarity and responsiveness while the core conceptual structure remained unchanged. The final interview guide is provided in Appendix 2.

All interviews were conducted by the first author (GÇN) in hospital offices or via secure online platforms, depending on participants’ preferences and institutional constraints. Of the fifteen interviews, six were conducted face-to-face, eight online, and one by telephone. No third parties were present during the interviews. Interviews were designed to last approximately 60 min to support sustained engagement while minimising participant fatigue. Participants were encouraged to elaborate on issues they considered important and were given time to reflect on their responses. Interview duration ranged from approximately 30 to 100 min.

In addition, interviews were audio-recorded with written consent. For one interview in which audio recording was not permitted, the researcher took detailed written notes. The participant subsequently reviewed and confirmed the documented responses. Field notes were maintained to document observational details, non-verbal cues, and emotional nuances relevant to the content of the interviews [35]. No repeat interviews were conducted.

### Data analysis

The analysis was conducted based on the interview transcripts and detailed interview notes. Two female researchers independently reviewed and analysed the interview records: GÇN (PhD), a specialist in the medical ethics, and formal training in qualitative research methods and RAE (PhD), a professor of the medical ethics with experience in qualitative health research.

Qualitative data analysis proceeded concurrently with data collection in an iterative manner [29]. Each interview was transcribed verbatim within two weeks of completion. Early transcription facilitated immersion in the data and enabled preliminary coding while subsequent interviews were still being conducted. Analysis began with repeated and immersive readings of the transcripts and field notes to achieve familiarity with the data and to identify meaning units relevant to the research aims [36]. Significant statements within the data were initially identified and coded. These initial codes were subsequently examined in relation to one another, and through iterative comparison, organised into coherent themes representing meaningful patterns across the dataset [37].

Although no substantial disagreements arose between the researchers regarding the identification of codes and themes, regular discussions were held throughout the analytic process to ensure a consistent interpretation of the data. In instances where alternative interpretations were possible, the researchers jointly reviewed the relevant transcripts to reach consensus [37].

The analytic process was also monitored through periodic consultations with two independent experts serving on the thesis committee: Professor with experience in qualitative research (MD) and Associate Professor of the medical ethics (PhD). These consultations provided additional methodological oversight and contributed to the credibility and transparency of the analytic process [38].

Themes are illustrated using verbatim participant quotations to preserve the authenticity of participants’ accounts and to enable readers to evaluate the relationship between the data and the analytic interpretations.

### Data handling and translation integrity

All interviews were conducted in Turkish. For this article, selected participant quotations were translated into English. Initial translations were undertaken by GÇN, who is fluent in both Turkish and English and familiar with the clinical and ethical dimensions of the data. The translated excerpts were subsequently reviewed by RAE to ensure linguistic accuracy and conceptual equivalence between the original and translated texts. Attention was paid to preserving participants’ intended meanings, contextual nuances, and ethical expressions. Where necessary, minor linguistic refinements were made to improve clarity and readability in English while maintaining fidelity to the original content.

### Researcher reflexivity

The interviews were conducted by GÇN, who has prior experience working in acute clinical environments alongside healthcare professionals, including emergency and surgical care settings, which provided familiarity with clinical culture and time-sensitive decision-making processes. However, the researcher had no prior professional or personal relationship with the participants before the study. This positioning allowed contextual understanding of clinical practice while reducing potential hierarchical influence during the interviews.

Reflexivity was maintained throughout the study as an ongoing methodological practice. The first author kept a reflexive journal during both data collection and analysis to document assumptions, emotional responses, and evolving interpretive perspectives. These reflections were regularly discussed with the second researcher to critically examine emerging interpretations and identify potential normative assumptions arising from the researcher’s background in medical ethics [39].

### Trustworthiness and rigour

Trustworthiness was ensured through strategies addressing credibility, dependability, and transferability in line with established qualitative research standards [38, 40]. Credibility was supported through prolonged engagement with the data, iterative discussions between the researchers during coding and theme development, and committee debriefing conducted during the analytic process. Dependability was enhanced by maintaining an audit trail documenting key methodological decisions, coding development, and reflexive notes throughout the research process. Transferability was supported through thick description, including detailed information about participants’ professional backgrounds, institutional settings, and the legal and organisational frameworks shaping neonatal decision-making in Türkiye. Together, these strategies strengthened methodological transparency and analytical rigor [40].

### Ethical considerations

Ethics approval was obtained from the Kocaeli University Non-Invasive Clinical Research Ethics Committee (no: 2022/04.12, date: 28 February 2022) and was conducted in accordance with the Declaration of Helsinki and relevant national data protection regulations (PDPL). Prior to participation, all neonatologists were provided with detailed written and verbal information about the aims and procedures of the study. Participation was entirely voluntary, and participants were explicitly informed of their right to withdraw from the study at any stage without providing a reason. Written informed consent was obtained from all participants before the interviews. Data were anonymised and stored securely, with access restricted to the research team. Confidentiality was strictly maintained throughout the processes of data collection, analysis, and reporting.

## Results

Four physicians who initially expressed interest withdrew prior to the interview stage due to time constraints or clinical workload, resulting in a final sample of 15 neonatologists. The participants (10 male, 5 female) were aged between 34 and 67 years (mean: 44 years) with professional experience ranging from 1 to 30 years (mean: 10 years). They were employed across university hospitals, training and research hospitals, and private healthcare institutions in different regions of Türkiye. Only one participant reported the availability of a formal ethics consultation service in their institution (Table 1).

**Table 1:** General characteristics of participants

Participant (*P*) no	Gender	Age (years)	Region	Years in profession	Years as neonatologist	Institution type	Availability of ethics support or institutional policies
1	Female	45	Marmara	20	12	Training& Research	No
2	Male	34	Marmara	8	1	Training & Research	No
3	Male	35	Marmara	12	2	Training & Research	No
4	Female	39	Marmara	15	4	Private	No
5	Male	48	Central Anatolia	22	9	Training & Research	No
6	Male	44	Marmara	22	14	Private	No
7	Female	47	Marmara	21	11	Private	No
8	Female	42	Marmara	18	8	Private	No
9	Male	43	Marmara	18	10	Private	No
10	Male	52	Marmara	27	8	University	No
11	Male	49	Black Sea Region	25	9	University	No
12	Male	50	Central Anatolia	27	10	Training & Research	No
13	Male	39	Marmara	16	3	Private	No
14	Male	47	Central Anatolia	24	17	University	No
15	Female	67	Marmara	43	30	University	Yes

Analysis of the qualitative data generated five interrelated themes that together capture neonatologists’ moral experiences of clinical decision-making in periviable care (Table 2). These themes reflect the epistemic, emotional, relational, and structural dimensions of clinicians’ moral experiences:

**Table 2:** Themes and subthemes of neonatologists’ moral experiences in periviable care

Main theme	Subtheme	Illustrative participant quotations
Moral decision-making under prognostic uncertainty	Prognostic uncertainty in the grey zone	“There are cases in between… Not every infant can be judged the same way. Whether they survive, or have neurological problems, it’s never completely clear.” (P11) “Between 22 and 24 weeks is the grey zone, that uncertain space. If you intervene, it’s a problem; if you don’t, it’s another problem.” (P14)
	Retrospective moral reflection	“You inevitably start questioning yourself at some point, wondering if doing it another way would have been better. You find yourself asking, ‘What am I doing?’” (P7)
Moral distress in perceived non-beneficial treatment	Preserving life versus avoiding harm	“It’s painful to see that it’s not helping anymore. Yet stopping feels like giving up. We’re trapped between the two.” (P10)
	Emotional desensitization	“At some point, I realized I wasn’t feeling sad anymore. That scared me—it felt like something inside me had died.” (P5)
Moral tensions in clinician–parent interactions	Balancing hope and realism	“Telling a family that their baby has a brain bleed and that the situation is bad is extremely difficult; it devastates them.” (P9)
	Delegation of decision-making responsibility	“In our country, families often adopt a more fatalistic attitude and tend to leave the responsibility to the physician, expecting treatment to continue.” (P9)
	Perceived inequities in care	“If an educated or wealthy family insists, we continue treatment longer; with poorer families, we wonder how they will cope. It shouldn’t be that way, but it happens.” (P7)
Legal and institutional constraints on ethical decision-making	Legal concerns shaping treatment decisions	“Sometimes it’s not only about the baby or the parents. There is also the hospital, the system, the fear of legal consequences. All of these influence what we do.” (P6)
	Professional hierarchy within clinical teams	“None of our senior physicians has ever told us not to intervene; within the hierarchy, the expectation is to continue treatment.” (P1)
	Limited institutional and legal guidance	There is no clear institutional or legal framework to guide these decisions for newborns. Because there are no clear guidelines or ethical counsel, we are forced to make these difficult decisions on our own.” (P14)
Moral loneliness in periviable care	Individual burden of responsibility	“Families often say, ‘You know best, doctor, do whatever you think is right.’ But that means every decision and its consequence are entirely yours.” (P6)
	Limited opportunities for shared ethical reflection	“There’s no ethics committee or guideline to consult. You make the decision, and you’re left alone with that weight.” (P9)
	Expectations of emotional resilience	“You must look strong. You can’t say, ‘This is too much for me.’ You just keep going as if nothing affects you.” (P10)

(i) moral decision-making under prognostic uncertainty,
(ii) moral distress in perceived non-beneficial treatment,
(iii) moral tensions in clinician–parent interactions,
(iv) legal and institutional constraints on ethical decision-making, and.
(v) moral loneliness in periviable care.

### Moral decision-making under prognostic uncertainty

Participants consistently described decision-making at the threshold of viability as a morally demanding process shaped by profound prognostic uncertainty. Prognostic indicators were perceived as insufficient to provide clear guidance regarding survival or long-term neurological outcomes. Several participants emphasized the difficulty of acting within the limits of medical knowledge. Although clinical indicators such as gestational age, birth weight, or early physiological responses could inform decisions, they were often regarded as probabilistic rather than determinative. This uncertainty meant that clinicians were required to make ethically significant decisions without being able to predict outcomes with confidence.

*“There are cases in between… Not every infant can be judged the same way. Whether they survive*,* or have neurological problems*,* it’s never completely clear.” (P11)*.

The gestational period between 22 and 24 weeks was frequently described as a “grey zone.” Participants used this term to capture the absence of clear clinical or ethical thresholds guiding intervention. Rather than representing a clinical category, the grey zone was experienced as a moral tension in which both intervention and non-intervention carried ethical consequences.

*“Between 22 and 24 weeks is the grey zone*,* that uncertain space. If you intervene*,* it’s a problem; if you don’t*,* it’s another problem.” (P14)*.

Importantly, participants indicated that uncertainty often extended beyond the moment of decision-making. Even after a clinical course had unfolded, neonatologists reported engaging in retrospective moral reflection, questioning whether alternative decisions might have been ethically preferable. This process of ongoing self-evaluation illustrated how uncertainty continued to shape clinicians’ moral experience even after the immediate clinical situation had passed.

*“You inevitably start questioning yourself at some point*,* wondering if doing it another way would have been better. You find yourself asking*,* ‘What am I doing?’” (P7)*.

### Moral distress in perceived non-beneficial treatment

Participants frequently described situations in which they continued intensive interventions despite perceiving that such interventions were unlikely to improve the infant’s condition in a meaningful way. Participants’ accounts revealed a deeper ethical tension between the professional responsibility to initiate or continue clinical interventions and the perception that ongoing interventions might prolong suffering or delay an inevitable outcome. A recurring component in these narratives was the simultaneous experience of recognising the limits of treatment while continuing life-sustaining interventions.

Neonatologists described moments when they believed that continued interventions were no longer clinically beneficial, yet withdrawing treatment appeared equally problematic due to due to perceived moral responsibility and the emotional weight.

*“Sometimes we know that what we’re doing no longer makes sense*,* but we can’t stop. Because stopping creates another kind of responsibility.” (P4)*.

Participants described feeling trapped between the desire to alleviate suffering and the professional commitment to provide care. This situation placed clinicians in a position where continuing or withdrawing life-sustaining treatment were both experienced as morally burdensome options.

*“It’s painful to see that it’s not helping anymore. Yet stopping feels like giving up. We’re trapped between the two.” (P10)*.

Over time, repeated exposure to such ethically difficult situations appeared to have cumulative emotional effects. Several participants reflected on how encountering similar dilemmas repeatedly could gradually erode their emotional responses.

*“At some point*,* I realized that I was no longer feeling sad. That frightened me; it felt as though something inside me had died.” (P5)*.

### Moral tensions in clinician–parent interactions

Participants emphasized that many of the most difficult moments in their practice emerged during interactions with parents. These encounters often generated moral tensions, as neonatologists sought to balance professional responsibility with sensitivity to parental hope, fear, and vulnerability.

Several participants described situations in which parents strongly wished for maximal intervention despite clinicians’ concerns about the infant’s prognosis. In these circumstances, clinicians felt an ethical obligation to respect parental perspectives while simultaneously grappling with their own professional judgment regarding the potential benefits and burdens of continued intervention. Navigating this tension required careful communication and emotional sensitivity, yet participants reported that such conversations were often fraught with emotional strain.

*“Parents want to hold on to hope*,* and we understand that. But sometimes we know that the chances are extremely small. Explaining this without destroying their hope is very difficult.” (P7)*.

Participants also reflected on the emotional weight associated with communicating uncertain or distressing information. The responsibility of conveying prognostic uncertainty while supporting families created a sense of moral pressure. Clinicians frequently described attempting to remain honest while also protecting parents from overwhelming despair.

*“You try to be transparent*,* but you also don’t want to crush them (families). Finding that balance is incredibly difficult.” (P11)*.

In addition to tensions surrounding communication and expectations about treatment, some clinicians described experiencing moral discomfort when parents appeared to place the ultimate responsibility for life-sustaining decisions entirely on the medical team. While participants recognized that families might feel unable to bear such decisions, assuming sole responsibility for choices involving life and death could create a profound sense of moral burden for clinicians.

*“Sometimes they say*,* ‘Doctor*,* you decide.’ But that makes it even heavier*,* because you know the weight of that decision stays with you.” (P2)*.

Participants also described situations in which families’ socioeconomic status or social influence appeared to shape treatment trajectories. In such cases, clinicians experienced ethical discomfort as they perceived that social power or family expectations could influence decisions about continuing intensive interventions. These situations were described as raising concerns about fairness and justice in clinical decision-making.

*“If an educated or wealthy family insists*,* we continue treatment longer; with poorer families*,* we wonder how they will cope. It shouldn’t be that way*,* but it happens.” (P7)*.

*“The baby’s father was politically influential. Even though we knew the chances were low*,* we continued all life-sustaining intervention because they demanded it.” (P11)*.

### Constrained ethical agency under legal and institutional pressures

Participants’ narratives suggested that moral challenges in periviable care were shaped not only by clinical uncertainty but also by broader institutional and structural conditions. Decision-making at the threshold of viability was described as occurring within a framework of legal accountability, professional hierarchies, and the absence of clear institutional ethical guidance.

Several neonatologists highlighted the influence of legal concerns on clinical practice. Participants described how the possibility of legal consequences could shape decisions about initiating or continuing treatment. In these circumstances, clinical actions were sometimes experienced as responses to perceived systemic pressures rather than the outcome of purely clinical or ethical deliberation.

*“Sometimes it’s not only about the baby or the parents. There is also the hospital*,* the system*,* the fear of legal consequences. All of these influence what we do.” (P6)*.

Participants also described how professional hierarchies could affect the expression of ethical concerns. Some clinicians suggested that junior physicians might hesitate to question decisions initiated by senior colleagues, even when they experienced ethical discomfort. Hierarchical dynamics were therefore perceived as limiting open discussion of ethically difficult treatment decisions.

*“You may feel that continuing treatment is not right*,* but if a senior physician has made the decision*,* it is difficult to openly challenge it.” (P8)*.

In addition, several neonatologists emphasised that the absence of clear institutional guidance for decision-making at the threshold of viability intensified the ethical burden placed on clinicians. Without formal ethical frameworks, consultative structures, or established protocols to guide these decisions, responsibility for navigating ethically complex situations was often perceived as resting heavily on the shoulders of individual practitioners.

*“There are moments when you wish there was a clearer framework or institutional support for these decisions. Otherwise*,* it feels like everything depends on you.” (P12)*.

### Moral loneliness in periviable care

Beyond clinical uncertainty and institutional constraints, participants described a profound sense of moral loneliness associated with decision-making at the threshold of viability. This loneliness was not experienced as physical isolation but rather as a moral and emotional solitude arising from the weight of responsibility attached to life-sustaining decisions for extremely premature infants. Participants frequently emphasized that even when decisions were discussed within teams, the moral consequences of those decisions were often felt at an individual level.

Several neonatologists noted that parents sometimes transferred responsibility for critical decisions entirely to the medical team. While such expressions were often interpreted as gestures of trust, clinicians described how this transfer of responsibility could intensify the moral weight of decision-making.

*“Families often say*,* ‘You know best*,* doctor*,* do whatever you think is right.’ But that means every decision and its consequence are entirely yours.” (P6)*.

Participants explained that in these situations, clinicians became the primary bearers of the moral responsibility associated with treatment decisions. Even when families were involved in discussions, neonatologists frequently felt that the final ethical burden remained with them.

This sense of moral solitude was further intensified by the limited opportunities for shared ethical reflection within clinical teams. Participants described how difficult decisions were often carried by individual clinicians rather than collectively processed within structured ethical discussions.

*“There’s no ethics committee or guideline to consult. You make the decision*,* and you’re left alone with that weight.” (P9)*.

Participants also reflected on how professional culture within clinical environments could discourage open expression of emotional or moral difficulty. Several neonatologists described an implicit expectation that physicians should remain composed and resilient, even in the face of emotionally demanding situations. This professional norm sometimes contributed to the invisibility of moral distress and emotional strain within the clinical environment.

*“You must look strong. You can’t say*,* ‘This is too much for me.’ You just keep going as if nothing affects you.” (P10)*.

## Discussion

This study explored how neonatologists in Türkiye experience the ethical dimensions of clinical decision-making at the threshold of viability. The findings indicate that periviable care is experienced not merely as a clinical challenge but as a complex moral practice shaped by uncertainty, relational responsibility, and institutional structures. Taken together, the results suggest that neonatologists’ experiences can be understood through three dimensions: epistemic burden in periviable decision-making, moral-emotional burden in treatment decisions and clinician–parent interactions, and structural constraints in periviable care.

### Epistemic burden in periviable decision-making

Participants consistently described decision-making in periviable care as occurring under conditions of profound prognostic uncertainty. The gestational period between 22 and 24 weeks was frequently described as a “grey zone,” where clinicians must make ethically consequential decisions despite limited predictive certainty regarding survival and long-term neurological outcomes. The concept of the grey zone is widely discussed in the neonatal ethics literature and has been conceptualised as an area in which treatment decisions may legitimately vary depending on clinical judgement, parental values, and contextual factors [2, 9, 41].

Within this zone, clinicians may be required to initiate or withhold life-sustaining interventions despite substantial uncertainty regarding both short-term survival and long-term neurodevelopmental outcomes, making decision-making at the threshold of viability one of the most ethically complex areas of neonatal medicine [2, 6].

However, the present findings suggest that uncertainty is not experienced merely as a limitation of medical knowledge but as a distinct moral burden. Participants described persistent self-questioning and retrospective doubt, reflecting the ethical weight attached to decisions made under uncertainty. Similar experiences have been reported in studies of neonatologists in Canada and Europe, where clinicians describe decision-making at the limits of viability as requiring them to assume responsibility for outcomes that cannot be reliably predicted in advance [6, 9]. In such circumstances, clinicians must act within the limits of available knowledge while bearing responsibility for decisions whose consequences may only become apparent over time [12].

Recent empirical work similarly emphasises that uncertainty in periviable care cannot be resolved through clinical evidence alone [6, 12, 41]. Contemporary analyses of neonatal decision-making highlight that even with improving survival statistics, prognostic variability remains substantial, leaving clinicians to interpret uncertain evidence in ethically meaningful ways [41]. Under such conditions, uncertainty becomes not only a matter of incomplete knowledge but also an epistemic condition in which clinicians must assume moral responsibility for decisions made under indeterminate outcomes [12].

The findings indicate that uncertainty in periviable decision-making is shaped not only by clinical considerations but also by the legal and institutional structures in which clinicians practise. Participants reported that national expectations to intervene when signs of life are present significantly influence clinical behaviour and may narrow the space for discretionary ethical judgement. In Türkiye, legal and regulatory arrangements, such as provisions of the Turkish Penal Code concerning potential liability for actions perceived as terminating life and administrative regulations requiring the registration of any infant showing signs of life as a live birth, may reinforce expectations for active intervention even under conditions of profound prognostic uncertainty [21, 22].

Within such regulatory environments, clinicians may feel compelled to consider not only what best serves the infant’s interests, but also which actions are least likely to expose them to legal or professional risk. This dynamic may shift the focus of decision-making from individualized ethical judgement toward legal defensibility and institutional compliance. Comparable patterns have been reported in other legally restrictive settings such as Greece [8, 13, 18] and Iran, where clinicians describe experiencing moral distress when institutional rules limit their capacity to align clinical actions with their ethical reasoning [17, 18]. Although concerns about potential legal liability were widely expressed by participants, only two reported experiences with legal proceedings—one involving the participant and another involving a colleague—and neither indicated that these cases resulted in criminal sanctions. This suggests that medico-legal concerns may operate more as a perceived risk influencing decision-making than as a frequently encountered legal outcome. As one participant noted, *“We may be more afraid than necessary.”*

Taken together, these findings suggest that epistemic uncertainty in periviable care is not experienced solely as an individual cognitive challenge but is embedded within broader legal and institutional environments that shape the range of ethically available actions [8, 12]. In this respect, clinicians’ experiences of uncertainty cannot be fully understood without considering the structural conditions that influence how ethical decisions are negotiated in practice [12]. This perspective provides an important basis for examining the structural constraints that shape periviable decision-making in Türkiye's healthcare system. 

### Moral-emotional burden in treatment decisions and clinician–parent interactions

In addition to epistemic uncertainty, participants described substantial moral-emotional strain associated with continuing life-sustaining interventions when the likelihood of meaningful recovery was perceived to be extremely limited. Clinicians’ concerns did not always reflect claims of strict quantitative futility; rather, participants often described situations in which ongoing interventions appeared unlikely to provide meaningful benefit while potentially prolonging suffering. This distinction reflects longstanding debates in bioethics regarding the interpretation of futility, which may refer either to the physiological inability of a treatment to achieve its intended biological effect or to broader concerns about whether continued intervention offers proportionate benefit relative to the burdens imposed on the patient [2, 42, 43].

Clinicians often described feeling caught between two ethically demanding responsibilities. On the one hand, they felt a strong professional obligation to preserve life; on the other hand, they expressed concern that continuing interventions might prolong the infant’s suffering or merely delay an inevitable outcome. Participants indicated that even when they believed that ongoing treatment no longer offered meaningful improvement, both continuing and discontinuing interventions could be experienced as morally burdensome choices. Neonatologists frequently described these situations as “prolonging the inevitable,” reflecting the tension between the desire to prevent further suffering and the fear that withdrawing interventions might be interpreted as abandoning care or could entail legal consequences. Such experiences are widely associated in the literature with moral distress, which arises when clinicians feel compelled to act in ways that conflict with their ethical judgement [10, 12].

International neonatal care guidelines emphasize that management options in such complex situations are not limited to the continuation of intensive life-sustaining interventions [44, 45]. In many European and North American healthcare systems, clinicians and parents may jointly consider options such as withholding, withdrawing, or limiting life-sustaining treatment [6, 7, 9, 46]. In such systems, care may shift toward palliative or comfort-focused approaches aimed at minimizing suffering and prioritizing the infant’s best interests and quality of life [47]. 

Within Türkiye's healtcare system, the absence of a clearly established legal framework governing the withholding, withdrawal, or limitation of life-sustaining treatment may generate considerable uncertainty and professional concern among clinicians. Although some participants recognised comfort-focused or palliative approaches as ethically appropriate when intensive treatment appeared disproportionate, structured neonatal palliative care pathways were reported to be rarely available in practice. One participant described neonatal palliative care as something that *“exists in name but not in practice.”* Participants suggested that several contextual factors contributed to this situation, including legal uncertainty surrounding treatment limitation and the absence of clear institutional guidance for implementing comfort-focused care. Previous studies from Türkiye similarly indicate that palliative care services in neonatal intensive care units remain underdeveloped and are often implemented informally rather than through established institutional frameworks [48].

Participants’ accounts suggest that clinicians rarely described making explicit decisions to withhold or withdraw treatment. Instead, they more commonly reported reassessing the intensity and scope of ongoing interventions, for example by avoiding further aggressive escalation or limiting the number and severity of invasive procedures when these were perceived as primarily prolonging suffering. However, even such adjustments were often accompanied by hesitation and ethical tension, reflecting the legal and institutional ambiguities surrounding end-of-life decision-making.

Importantly, this ethical tension was not experienced solely because of clinical judgment. Within the legal and institutional framework in Türkiye, the normative expectation of life conservation is reinforced not only by cultural and moral values ​​but also by institutional and policy frameworks that encourage active intervention when signs of life are detected [21, 22]. Participants’ narratives show that this structural and normative conditions frequently intersect with deeply ingrained beliefs about the sanctity of life. Many neonatologists reported feeling a strong moral obligation to intervene when signs of life are detected. At the same time, clinicians acknowledged that continued interventions in certain clinical courses may prolong the infant’s suffering and may not provide realistic expectations for recovery. This creates a profound ethical tension between the duty to conserve life and the obligation to avoid harm.

Participants frequently described experiencing tension between the professional duty to preserve life and the ethical concern that ongoing interventions might prolong suffering or delay an inevitable outcome. Similar experiences have been documented among NICU professionals internationally, where clinicians report moral distress when they feel obliged to continue interventions that conflict with their ethical judgement regarding the infant’s best interests [9, 10, 12].

Comparable ethical tensions have been reported in neonatal intensive care settings in other healthcare systems. Studies examining clinicians’ perspectives on the management of extremely preterm infants also highlight the complexity of decision-making at the threshold of viability [49]. For example, studies conducted in Israel show that decisions regarding resuscitation at the threshold of viability generate a strong sense of personal ethical responsibility among neonatologists [50]. Similarly, research from Greece shows that neonatologists frequently experience ethical conflict when balancing life-saving interventions with concerns about the infant’s future quality of life [8, 13, 18]. In addition, studies from Iran suggest that cultural and religious values may further reinforce clinicians’ tendency to pursue life-supporting treatment even in ethically challenging situations [19]. These studies demonstrate that tensions between life-saving and harm-avoidance constitute a broader ethical challenge across diverse healthcare systems, including settings such as Türkiye.

Relational dynamics with parents further intensified these experiences. Participants described considerable difficulty communicating prognostic uncertainty while attempting to preserve parental hope and emotional stability. Previous research similarly indicates that discussions with families at the threshold of viability represent one of the most emotionally demanding aspects of neonatal practice [6, 51]. Communicating uncertain prognoses while maintaining honesty and empathy therefore emerged as a central moral challenge within clinician–parent interactions.

Participants also described situations in which parents transferred decision-making responsibility entirely to clinicians. Although such expressions were often interpreted as trust, clinicians experienced this transfer as intensifying the moral weight of their decisions. These findings resonate with broader ethical discussions concerning shared decision-making in neonatal care, which emphasise collaborative deliberation between clinicians and parents when determining the most appropriate course of action for periviable infants [41]. However, the experiences described in this study suggest that achieving such collaboration may be difficult in practice. When parents defer decisions entirely to clinicians, the ideal of shared decision-making may shift toward a form of asymmetric responsibility, where clinicians ultimately carry the ethical burden of decisions concerning life-sustaining treatment.

In neonatal ethics, decision-making for infants differs fundamentally from adult medical decision-making models centered on individual autonomy. Because newborns cannot exercise autonomous choice, decision-making is typically grounded in parental authority and professional responsibility. The central ethical framework guiding such decisions is the best interest standard. Kopelman conceptualizes this standard not as a single rule but as a multifaceted framework functioning as an intervention threshold, an ethical ideal, and a standard of reasonableness in clinical decision-making [52]. Accordingly, clinicians and parents often seek not a single objectively “best” outcome but rather an ethically acceptable course of action that reasonably promotes the infant’s welfare among available options. However, the findings of this study indicate that several participants described experiencing a substantial responsibility in contributing to and guiding decisions regarding the infant’s care. In situations involving extremely poor prognosis, this responsibility was perceived by some clinicians as a significant moral burden.

In addition to these relational and communicative challenges, participant responses suggested ethical tensions related to social inequality in clinician–parent encounters. In some situations, clinicians perceived that families with greater socioeconomic resources or political influence were able to exert stronger pressure for maximal treatment despite concerns about prognosis. Conversely, families with fewer resources were more likely to defer decision-making responsibility to clinicians, particularly when concerns regarding long-term disability or caregiving burden were raised. Some participants also acknowledged that awareness of a family’s anticipated capacity to care for a child with severe disability could influence their own clinical reflections, occasionally generating further ethical discomfort. Similar observations have been reported in neonatal ethics literature, where clinicians describe how differences in parental resources, educational background, or social influence may affect how strongly treatment preferences are expressed and negotiated within clinical encounters [6, 9]. These findings suggest that the moral–emotional burden experienced by clinicians in periviable care arises not only from prognostic uncertainty but also from the ethical responsibility of balancing multiple competing considerations, including preserving life, minimising suffering, respecting parental perspectives, and maintaining fairness in clinical decision-making. The cumulative weight of these responsibilities may have important emotional consequences for clinicians over time.

Participants described how repeated exposure to such ethically challenging situations could gradually lead to emotional erosion. Some clinicians reported developing emotional distancing as a way of maintaining professional functioning under sustained moral pressure. Similar patterns have been documented among NICU professionals in Canada, Switzerland, and Greece, where repeated exposure to ethically complex decisions has been associated with emotional withdrawal, coping through adaptive distancing, and increased risk of burnout [6, 8, 10].

### Structural constraints in periviable care

Perhaps one of the central contributions of this study lies in its depiction of the structural conditions shaping clinicians’ moral experiences. Beyond clinical uncertainty and relational tensions, participants described decision-making at the threshold of viability as occurring within institutional environments characterised by legal accountability, professional hierarchies, and limited access to formal ethical consultation. Participants emphasised the limited availability and accessibility of structured mechanisms for ethical deliberation in neonatal care settings. Ethics committees were rarely accessible for clinical consultation, and institutional guidance regarding treatment limitation or neonatal palliative care was perceived as limited or unclear. Studies examining the structure and functioning of ethics committees in Türkiye have similarly highlighted variability in their accessibility and practical role in clinical decision-making [53, 54]. In the absence of accessible and structured mechanisms for ethical deliberation, responsibility for ethically significant decisions was frequently experienced as resting on individual clinicians. Although decisions were often discussed informally within clinical teams, such discussions did not fully mitigate this concentration of responsibility. Similar patterns have been reported in healthcare settings where limited institutional support leads clinicians to internalise moral responsibility rather than share it collectively [55, 56].

Building on this, participants’ narratives further suggested that these structural conditions do not merely frame decision-making but actively shape how responsibility is experienced in practice. Rather than being distributed across teams or institutional processes, ethical responsibility was frequently experienced as concentrated at the level of individual clinicians. In neonatal care, decision-making has been described as complex and context-dependent, particularly under conditions of uncertainty [57]. This concentration ethical responsibility may be further intensified by professional cultures that discourage open discussion of emotional vulnerability. Participants described implicit expectations that physicians remain composed and resilient regardless of emotional strain, potentially rendering moral distress less visible within clinical environments. These conditions give rise to experiences consistent with moral loneliness, in which clinicians bear the ethical weight of consequential decisions without structured opportunities for shared deliberation or institutional support [12].

Comparable ethical tensions have been documented in neonatal intensive care settings across different healthcare systems. A qualitative study conducted in Israel indicates that decisions regarding resuscitation at the threshold of viability generate a strong sense of personal ethical responsibility among neonatologists [50]. Research from Greece similarly demonstrates that conflicts between obligations to preserve life and concerns regarding quality of life create significant ethical tensions in neonatal end-of-life decision-making [8, 13, 18]. In Switzerland, disagreements regarding the extent of parental involvement have also been identified as a source of ethical tension among neonatal care professionals [58]. While these studies primarily describe experiences of moral distress or ethical conflict, the findings of the present study suggest that, in contexts where institutional support mechanisms remain limited, such tensions may also be experienced as moral loneliness. This reinforces the view that clinicians’ moral experiences are embedded within institutional and organisational conditions that shape how ethical responsibility is distributed.

Evidence from clinical ethics and neonatal care settings suggests that structured forms of ethical support have been shown to support shared deliberation and improve clarity in complex decision-making. Clinical ethics consultation services and ethics committee involvement have been associated with improved clarity in decision-making processes and more structured ethical deliberation in complex clinical cases [56, 59]. In neonatal intensive care specifically, interdisciplinary collaboration models—such as regular joint discussions between neonatology and palliative care teams—have been shown to support shared decision-making and reduce the perceived burden of ethically complex decisions [60].

At the same time, the relationship between formal guidance and clinical practice remains complex. Evidence from the United Kingdom indicates that the presence of national frameworks does not necessarily ensure alignment with clinical practice, particularly under conditions of prognostic uncertainty [61]. This suggests that while guidelines and institutional mechanisms are important, their effectiveness depends on how they are implemented and integrated within clinical environments.

The findings of this study suggest that neonatologists’ experiences in periviable care are shaped by the interaction of epistemic, emotional, and structural burdens. Moral distress and moral loneliness may be understood not solely as individual responses to difficult decisions, but as experiences shaped by organisational environments and the distribution of ethical responsibility within healthcare systems [12, 55, 57].

### Limitations

This study provides an in-depth qualitative exploration of neonatologists’ moral experiences in periviable care within Türkiye's healthcare system. By adopting a phenomenological approach, the study captures how clinicians interpret and navigate ethically complex decisions under conditions of prognostic uncertainty, relational responsibility, and institutional constraints.

Several limitations should nevertheless be acknowledged. The study involved a purposive sample of neonatologists who volunteered to participate, which may introduce selection bias and may limit the transferability of the findings. As with all qualitative research, the results reflect participants’ subjective experiences and interpretations rather than objective measurement. In addition, the study focused primarily on clinicians’ perspectives; future research incorporating parental experiences and multidisciplinary team perspectives could provide a more comprehensive understanding of decision-making processes at the threshold of viability. Finally, although interviews were conducted in Turkish and carefully translated into English with attention to conceptual equivalence, some linguistic or cultural nuances may not have been fully preserved.

## Conclusion

This study shows that decision-making at the threshold of viability is experienced by neonatologists not only as a clinical challenge but also as a complex moral practice shaped by uncertainty, relational responsibility, and institutional conditions. Clinicians’ experiences reflect a cumulative moral burden produced by the interaction of prognostic uncertainty, emotionally demanding treatment decisions, clinician–parent interactions, and structural constraints within the healthcare system.

By demonstrating how these dimensions intersect in everyday clinical practice, the study contributes to a more nuanced understanding of the ethical realities of periviable care. These findings highlight the phenomenon of moral loneliness, showing how limited institutional ethics support can intensify clinicians’ sense of individual moral responsibility in ethically complex situations.

These findings have important implications for neonatal care practice and policy. They suggest that moral distress and moral loneliness should be recognised not only as individual experiences but as outcomes shaped by organisational and systemic conditions. Strengthening institutional support structures such as ethics consultation services, interdisciplinary case deliberation forums, and communication training may facilitate shared ethical reflection and reduce the concentration of responsibility on individual clinicians. In addition, national and institutional guidance regarding periviable care, including frameworks for treatment limitation and neonatal palliative care, may support more consistent and ethically grounded decision-making in periviable care.

## Supplementary Information

Supplementary Material 1.

Supplementary Material 2.

## Data Availability

The data used and/or analysed during the current study are available from the corresponding author on reasonable request.

## Abbreviations

COREQ: Consolidated Criteria for Reporting Qualitative Research
NICU: Neonatal Intensive Care Unit
PDPL: Personal Data Protection Law
