# Supplementary material for: At the edge of viability: neonatologists’ moral experience under uncertainty in Türkiye
Source: BMC Med Ethics. 2026 Jun 12;27:167. doi: 10.1186/s12910-026-01466-8 (PMC13483495; doi:10.1186/s12910-026-01466-8)
Supplement: Supplementary file 2 [file 12910_2026_1466_MOESM2_ESM.pdf]

Appendix 2

Interview Guide

Interview number:
Date of interview:
Place of interview:
Start time of the interview:
End time of the interview:

Participant Information and Consent

- Briefly introducing the purpose of the interview.
- Explaining the research information form.
- Obtaining written informed consent.
- Asking if the participant has any questions.
- Requesting permission to record the interview.

Section 1:

Socio-Demographic and Professional Characteristics

- Can you provide some information about yourself?
- Name (for addressing purposes)
- Gender
- Age
- Marital status
- Number of children (if any)
- Status and duration of ethics training
- Specialist training in veterinary medicine
- Duration of working in the profession
- Duration of working with experimental animals
- Certificates and training you have received for work with experimental
-

Section 2:

Warm-Up Questions

- How do you manage the medical decision-making process for an extremely preterm infant (especially between 22–25 weeks of gestation)?
- What criteria do you prioritize when making medical decisions for these infants?

Possible Opening Questions

- Have you handled a case involving an infant born at less than 24 weeks of gestation?
- If yes, what factors facilitated medical decision-making in this case?
- What factors made decision-making more challenging?
- What could have made this case easier or more difficult to manage?

Case-Specific Questions:

- Could you briefly describe the case? (Based on their response, follow up with detailed questions to explore the context.)
- What was your role in the medical decision-making process?
- What was the primary challenge in making medical decisions for this case?
- What factors complicated the decision-making process?

- What factors made decision-making easier?
- What decision was ultimately made?
- Were alternative decisions considered?
- How did you feel during the decision-making process?
- How did the parents react to the decisions made?

Ethical Considerations

- Did you perceive any ethical challenges in this case?
- What was the most significant ethical issue?
- Do you think this case involved resource allocation concerns?
- Do you believe the decision made was the right one?
- Would you make the same decision again, or would you act differently in any way?

Role of Parents

- What role did the parents play in medical decisions for their infant's treatment?
- In your opinion, should parents have been more or less involved in the decision-making process?
- Were there differences between your decisions and those of the parents?
- If so, what were they?

Clinical Decisions for Extremely Preterm Infants

- There are no clear guidelines for medical interventions for infants born between 22 and 25 weeks of gestation, creating a "gray zone" in decision-making.
- Do you approach decision-making differently for infants within and outside this gray zone?
- If so, what are the main differences?
- What factors make decision-making for infants in the gray zone more difficult?

Possible Closing Questions

- Does your institution have policies or guidelines to assist neonatologists with medical decisions for extremely preterm infants?
- If yes, what are your views on these policies? Do you agree with them?
- Does your institution have an ethics committee or ethical expert available for consultation on such cases?
- Do you apply the recommendations provided by the ethics committee or expert?
- If invited by your hospital administration to contribute to developing institutional policies on medical decisions for extremely preterm infants, what would your main recommendations be?
- If you were advising a younger colleague on medical decision-making for extremely preterm infants, what advice would you give?

Additional Questions:

- Is there anything else you would like to share regarding this topic?

Interview Notes

[Space for additional comments, observations, or details noted during the interview.]
